# N- and O-glycosylation patterns and functional testing of CGB7 versus CGB3/5/8 variants of the human chorionic gonadotropin (hCG) beta subunit

**DOI:** 10.1007/s10719-020-09936-w

**Published:** 2020-08-07

**Authors:** Karina Biskup, Véronique Blanchard, Paola Castillo-Binder, Henry Alexander, Kurt Engeland, Sindy Schug

**Affiliations:** 1 Charité Universitätsmedizin Berlin, Corporate Member of Freie Universität Berlin, Humboldt-Universität zu Berlin, and Berlin Institute of Health, Institute of Laboratory Medicine, Clinical Chemistry and Pathobiochemistry, Augustenburger Platz 1, 13353 Berlin, Germany; 2 Division of Molecular Oncology and Division of Human Reproduction and Endocrinology, Department of Obstetrics and Gynecology, Medical School, University of Leipzig, Semmelweisstr. 14, 04103 Leipzig, Germany

**Keywords:** CGB7, Human chorionic gonadotropin, hCG, Glycan structure, ERK pathway, Differential expression, Glycosylation, Pregnancy, p53, Recombinant proteins

## Abstract

The classical function of human chorionic gonadotropin (hCG) is its role in supporting pregnancy. hCG is a dimer consisting of two highly glycosylated subunits, alpha (CGA) and beta (CGB). The beta-hCG protein is encoded by *CGB3, CGB5, CGB7* and *CGB8* genes. *CGB3*, *5* and *8* code for an identical protein, CGB3/5/8, whereas CGB7 differs in three amino acids from CGB3/5/8. We had observed earlier that CGB7 and CGB3/5/8 display very distinct tissue expression patterns and that the tumor suppressor and transcription factor p53 can activate expression of *CGB7* but not of *CGB3/5/8* genes. Here, we investigate the glycan structures and possible functional differences of the two CGB variants. To this end, we established a system to produce and isolate recombinant CGA, CGB7 and CGB3/5/8 proteins. We found that N- and O-glycosylation patterns of CGB7 and CGB3/5/8 are quite similar. Functional assays were performed by testing activation of the ERK1/2 pathway and demonstrated that CGB7 and CGB5/5/8 appear to be functionally redundant isoforms, although a slight difference in the kinetics of ERK1/2 pathway activation was observed. This is the first time that biological activity of CGB7 is shown. In summary, the results lead to the hypothesis that CGB7 and CGB3/5/8 do not hold significant functional differences but that timing and cell type of their expression is the key for understanding their divergent evolution.

**Electronic supplementary material:**

The online version of this article (10.1007/s10719-020-09936-w) contains supplementary material, which is available to authorized users.

## Introduction

The glycoprotein hormone human chorionic gonadotropin (hCG) is known for its pregnancy-supporting role [1, 2]. It is produced mainly by trophoblast cells. Interestingly, we observed that hCG is also produced by the secretory endometrium at the time of implantation [3–5]. Its classical endocrine function is to maintain progesterone production by the corpus luteum, but in recent years various additional effects, mainly paracrine, have been described. For example, hCG is important to induce immune tolerance at the fetal-maternal interface as it promotes decidualization and angiogenesis [1, 2, 6–12]. It is therefore essential for embryo implantation. In general, hCG is indispensable for the establishment and maintenance of early human pregnancy.

The hCG protein complex is composed of two non-covalently linked subunits, alpha (CGA) and beta (CGB). Both subunits are highly glycosylated. Its alpha subunit is common to luteinizing hormone (LH), follicle-stimulating hormone (FSH) and thyrotropin (TSH), whereas the respective beta-subunits confer the biological specificities. hCG acts mainly via a G protein-coupled receptor named Lutropin-choriogonadotropic hormone receptor (LHCGR) that it employs together with LH [2, 13]. Downstream of LHCGR, multiple signaling enzymes are activated, including adenylyl cyclase and inositol phospholipid-specific phospholipase C, *e.g*. leading to the activation of ERK1/2 kinases [14].

Interestingly, four different genes were found to code for beta-hCG (CGB) proteins. All four proteins, CGB3, 5, 7 and 8 are relevant for pregnancy. *CGB3*, *5* and *8* genes code for identical proteins whereas the often neglected CGB7 isoform differs in three amino acids from CGB3/5/8 [15, 16]. We observed that there is a distinct gene expression pattern between *CGB7* and *CGB3/5/8*. While CGB3/5/8 protein is produced in large amounts by the trophoblast, the luteal phase endometrium solely secretes the CGB7 variant protein [5]. Additionally, we detected CGB7 in other epithelial tissues like decidua, urothelium and retina [17–21] and found that the tumor suppressor and transcription factor p53 specifically induces the expression of *CGB7* by direct binding to its promoter. The other *CGB* variants do not contain the identified p53 consensus element in their promoters and are unaffected by p53 activation [22].

So far, not much attention was dedicated to CGB7, as the expression levels of *CGB3, 5 and 8* genes were shown to be higher than that of *CGB7* in placenta and choriocarcinoma [16]. All previous studies investigating hCG activity used either recombinant hCG with only CGB3/5/8 or mixed hCG preparations purified *e.g*. from pregnancy urine. Considering the clearly differing expression patterns of the *CGB* variants, it was the aim of this study to investigate further differences of the resulting CGB protein variants. As 30% of CGB’s mass is made of N- and O-glycan chains, it is essential to compare the glycosylation patterns of CGB7 and CGB3/5/8, particularly as these alterations may result in different functional activities. Several studies have already addressed glycan analysis of hCG [23–29]. However, the distinct CGB variants were not yet analyzed separately. Although the three amino acids by which CGB7 and CGB3/5/8 differ are not glycosylated themselves, some of them are located in close proximity to glycosylated residues and therefore may affect interactions with glycosylating enzymes [30]. To study the glycan structures of the CGB variants, we generated HEK293 and HeLa cell lines stably expressing either CGB7 or CGB3/5/8 with His-tags for purification. N- and O-glycans of hCG from different single cell clones as well as from cell pools were analyzed.

Whether the hCG variants exert individual functions had also not been addressed yet. To this end, we created stably transfected cell lines expressing the individual CGB variants together with CGA alpha subunit to test for ERK1/2 activation as a functional test.

## Results

In this study, we aimed at elucidating possible different glycosylation patterns and associated divergent functions of the hCG subunits CGB7 and CGB3/5/8.

### Production of recombinant hCG subunits CGB7, CGB3/5/8 and CGA

**Fig. 1:**
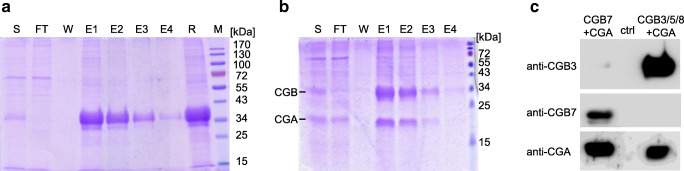
Recombinant expression and purification of hCG subunits. Recombinant proteins were expressed in stably transfected HEK293 cells and isolated by affinity purification. (A + B) SDS-PAGE followed by Coomassie staining of affinity purification samples: S = cell culture supernatant before purification; FT = flow through; W = wash fraction; E1–4 = elution fractions; R = E1 + 2 after rebuffering and concentration with VivaSpin® columns. 20 μl per lane were applied except for R (5 μl), M = molecular weight marker. Affinity purification of recombinant **a** CGB3/5/8 and **b** CGB3/5/8 + CGA. **c** Purified supernatants of HEK293 cells stably expressing CGB7 or CGB3/5/8 with CGA were analyzed by Western blot using anti-CGB3, anti-CGB7 and anti-CGA antibodies. Cells transfected with empty pcDNA3.1(+)-His vector served as negative control (ctrl). All samples were prepared under reducing conditions yielding monomeric subunits. These samples were run on the same blot, which was then cut and incubated with the respective antibodies

In order to obtain specific preparations of the two CGB variants free of the other variant, recombinant CGB7 and CGB3/5/8 were produced. To this end, HEK293 as well as HeLa cells were stably transfected with plasmids coding for the CGB7 and CGB3/5/8 isoforms to create cell preparations that express one of the two variants. HEK293 cells were chosen because they allow good yields for recombinant protein expression. HeLa cells were selected to analyze a second cell line, preferably from the human reproductive tract. Recombinant CGB was secreted into the supernatant and purified by nickel-histidine affinity chromatography. Purification efficiency as well as CGB specificity were controlled by SDS-PAGE and Western blotting, respectively (Fig. 1). Figure 1a shows one representative analysis of the CGB affinity purification fractions. As expected, elution fraction E1 contained most of the recombinant CGB.

For functional studies on ERK1/2 activation, synthesis and purification of dimeric hCG consisting of CGA and CGB was required. To this end, we created double-stably transfected HEK293 cell lines, expressing CGB7 as well as CGA or CGB3/5/8 with CGA. Purification of dimeric hCG was carried out as described for CGB single subunits. Co-purification of CGA with CGB subunits was controlled by SDS-PAGE (Fig. 1b). Western blotting confirmed co-expression of CGA and verified the identity of recombinant CGB variants using our custom-made isoform-specific antibodies (Fig. 1c).

### N- and O-glycan analysis of hCG variants

**Fig. 2:**
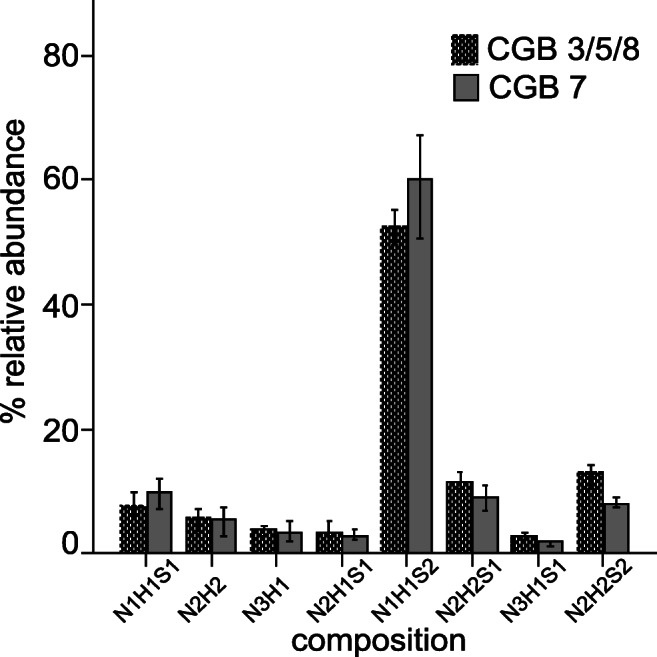
Median values of relative areas of all O-glycans bound to CGB3/5/8 and CGB7 expressed in HEK293 cells. The bar chart was generated by a median of all measurements (*n* = 4) performed. The error bars indicate 95% CI (confidence interval)

Biological replicates were analyzed for each cell line, namely *n* = 4 for HEK293 and *n* = 2 for HeLa cells. N- and O-glycans were cleaved enzymatically and chemically from CGB, extracted from the SDS-PAGE gel and permethylated followed by measurements by MALDI-TOF mass spectrometry. 86 N-glycan structures (Supplementary Table S1, Supplementary Figs. S1 and S2) and 8 O-glycan structures were identified (Fig. 2, Supplementary Fig. S3, Supplementary Table S2). N-Glycans were mostly of the complex-type but traces of high-mannose 5 (*m/z* 1579.8) and hybrid structures (*m/z* 2173.1, 2186.1, 2360.2, 2390.2) were detected as well. For CGB expressed in HEK293 cells, O-glycans consist of the mono- and disialylated structures H1N1S1 and H1N1S2, which are likely to be of the core 1 type as well as larger structures, namely H2N2, H2N2S1, H2N2S2, H1N3 and H1N3S2, which are likely to be of the core 2 type. The low amounts of hCG obtained from HeLa cells prevented the detection of O-glycan signals in the MALDI-TOF mass spectra.

### Glycosylation of CGB produced in HEK293 cells

CGB produced in HEK293 cells was bearing mostly biantennary neutral and sialylated fucosylated N-glycans carrying GalNAc, which is a hallmark of N-glycosylation in HEK293 cells: (*m/z* 2500.3, 2646.3, 2805.4) [31]. Digalactosylated structures were also present at *m/z* 2605.3, 2779.4, 2966.5 and tri-antennary structures were found as traces. MALDI-TOF/TOF fragmentation of the most abundant structures was carried out to verify N-glycan structures. In Supplementary Fig. S4, the following diagnostic fragments demonstrate the presence of GalNAc in CGB produced in HEK293 cells: *m/z* 643.3 (N1S1) and 888.4 (N2S1) in the parent *m/z* 3007.5 (Fig. S4A), *m/z* 527.3 (N2) and 905.4 (F1N2S1) in the parent *m/z* 2500.3 (Fig. S4C). Core-fucosylation was proven with the diagnostic fragments *m/z* 474.2 (Fig. S4A-C) and 719.3 (Fig. S4A). Difucosylation occurred in biantennary structures, one fucose being located at the core and the other one at an antenna as shown by the presence of a non-fucosylated antenna at *m/z* 847.4 (F1H1N1) in the parent *m/z* 2820.4 (Fig. S4B) and at *m/z* 527.3 in the parent *m/z* 2500.3 (Fig. S4C).

### CGB glycosylation purified from HeLa cells

CGB from HeLa cells was decorated with a high proportion of biantennary core-fucosylated N-glycans (*m/z* 2605.3, 2779.4, 2966.5). By comparison with HEK293 cells, CGB from HeLa cells was more sialylated. The mono- and disialylated biantennary digalactosylated structures at *m/z* 2605.3 and 2966.5, respectively, were core-fucosylated as shown by the diagnostic fragments at *m/z* 474.2, 719.4, 2154.0 and 2515.2 (Figs. S5A and S5C). In the MALDI-TOF/TOF spectrum of the difucosylated N-glycan at *m/z* 2779.4 (Fig. S5B), two isomers were identified, the first one having both core and antennary fucosylation (diagnostic fragments at *m/z* 474.2, 847.4, 1486.7, 1690.8, 1955.0, 1971.0, 2328.1) and the second one bearing solely antennary fucosylation (diagnostic fragments at *m/z* 939.4, 1143.5, 1592.8).

### Comparison of the N-glycome of CGB3/5/8 and CGB7

**Fig. 3:**
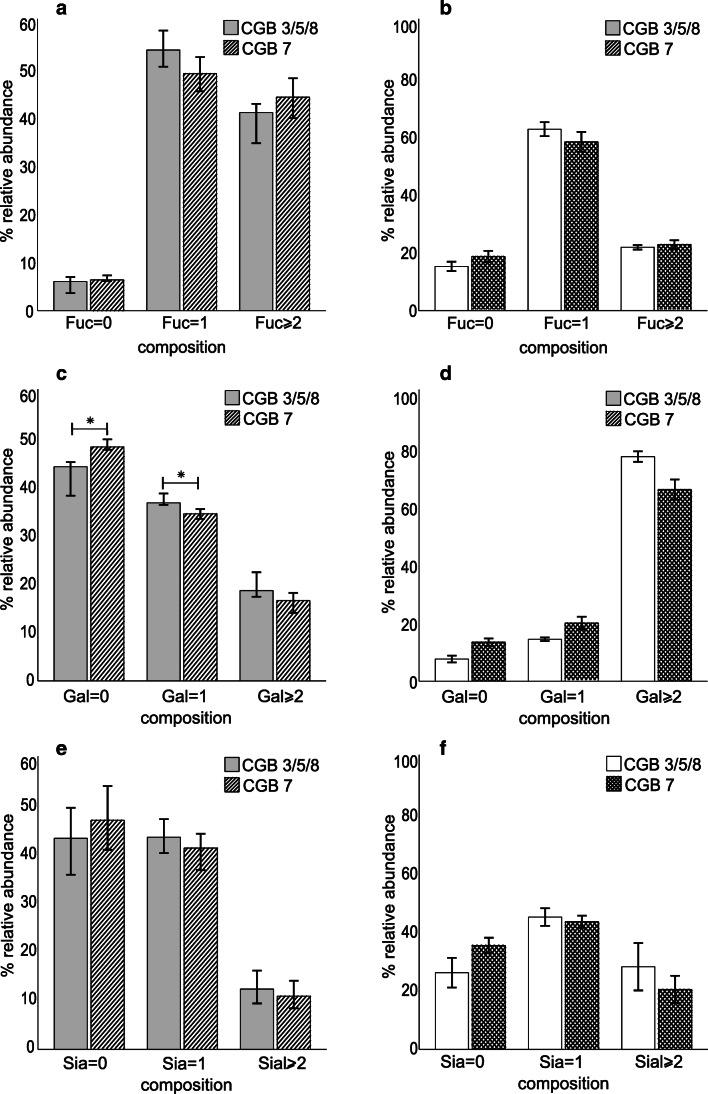
Median values of relative areas of N-glycans bound to CGB3/5/8 and CGB7 expressed in (**a**, **c**, **e**) HEK293 and (**b**, **d**, **f**) HeLa cells as analyzed by MALDI-TOF-MS. *p < 0.5, **p < 0.01. (**a**, **b**) N-Glycan fucosylation; (**c**, **d**) N-glycan galactosylation; (**e**, **f**) N-glycan sialylation. Bar charts were generated by a median of the sum of relative intensities of glycan structures from all experiments performed (*n* = 4 for HEK293 cells and *n* = 2 for HeLa cells). The error bars indicate 95% CI (confidence interval)

The N-glycan profiles of CGB3/5/8 and CGB7 expressed in HEK293 and HeLa cells were compared using the median relative N-glycan intensities from four and two independent experiments, respectively. Glycan profiles were qualitatively similar but small differences were observed as described in the following. Fucosylation was a little higher in CGB7 than in CGB3/5/8 in HEK293 cells (Fig. 3a) and sialylation was slightly higher in CGB3/5/8 than in CGB7 both in HEK293 and HeLa cells although there was no statistical significance (Fig. 3e, f). CGB7 was less galactosylated than CGB3/5/8 for both cell types and the difference was statistically significant in HEK293 cells (Fig. 3c, d).

### Induction of ERK1/2 phosphorylation by hCG variants containing CGB3/5/8 or CGB7 subunits

**Fig. 4:**
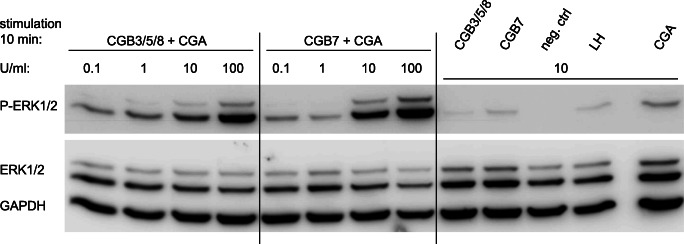
Phosphorylation of ERK1/2 after stimulation with different concentrations of hCG variants. HEK293 cells stably expressing LHCGR were stimulated with hCG containing CGA with CGB7 or CGB3/5/8, respectively. Phosphorylated ERK1/2 (P-ERK1/2), total ERK1/2 and GAPDH protein levels were analyzed by Western blot (*n* = 3 individual experiments; representative blots are shown). Cells were stimulated for 10 min with different concentrations of hCG. Single subunits and recombinant LH served as controls. One blot was initially incubated with anti-P-ERK1/2 (upper graph), then stripped and incubated both with anti-ERK1/2 and GAPDH antibodies (lower graph)

**Fig. 5:**
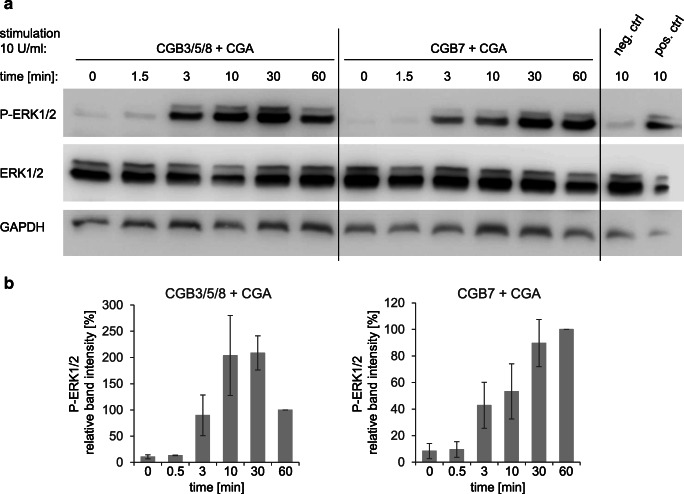
Kinetics of ERK1/2 phosphorylation after stimulation with hCG variants. HEK293 cells stably expressing LHCGR were stimulated for various times with 10 U/ml of hCG containing CGA with CGB7 or CGB3/5/8, respectively. **a** Phosphorylated ERK1/2 (P-ERK1/2), total ERK1/2 and GAPDH protein levels were analyzed by Western blot (*n* = 3 individual experiments; representative blots are shown). A commercially available recombinant hCG preparation served as positive control. Two separate blots were processed in parallel from the same samples with the same quantities. **b** Densitometric analysis of P-ERK1/2 normalized to total ERK1/2 band intensities is displayed in percent. Relative band intensity after 60 min of stimulation was set to 100%

Human chorionic gonadotropin is known to activate, amongst others, the ERK1/2 pathway via the LH/hCG receptor (LHCGR) [14]. As a result, ERK1/2 proteins are phosphorylated. To test whether our recombinant dimeric hCG variants consisting of CGA with CGB7 or CGB3/5/8 subunits are functional and if the different CGB proteins vary in their ability to induce this signal transduction pathway, we detected phosphorylated ERK1/2 (P-ERK1/2) by Western blotting after stimulation with hCG (Figs. 4 and 5). To this end, we used HEK293 cells stably expressing LHCGR. Stimulation by hCG was carried out employing different hCG variant concentrations (Fig. 4) or by incubating for different time spans (Fig. 5), respectively. Total ERK1/2 and GAPDH protein levels were analyzed as controls. Recombinant hCG leads to a clear phosphorylation of ERK1/2 using 10 U/ml of hCG (Fig. 4). With 100 U/ml, phosphorylation of ERK1/2 was even more pronounced. Remarkably, both hCG variants are able to induce ERK1/2 phosphorylation. This is the first time that CGB7 was shown to be functionally active. A significantly different activation of the ERK1/2 pathway by dimeric hCG containing CGB7 or CGB3/5/8 was not observed. As controls, the figure also shows that single CGB or CGA subunits as well as LH did not induce ERK1/2 phosphorylation. Using 10 U/ml of hCG, we then stimulated the cells for different periods of time. A slight difference between hCG containing CGB3/5/8 or CGB7 was observed: With CGB3/5/8 the time course of P-ERK1/2 protein concentration levels appeared to be somewhat shifted to earlier time points compared with CGB7 (Fig. 5a). To verify this, we conducted a semi-quantitative densitometric analysis of band intensities of P-ERK1/2 normalized to total ERK1/2, confirming a slight difference in kinetics of ERK1/2 activation (Fig. 5b).

The major observation is that both types of recombinant hCG variants are functionally active. Even with small differences in active concentrations and kinetics, both, CGB7 as well as CGB3/5/8 proteins, are generally able to activate the ERK1/2 pathway.

## Discussion

It has long been of interest why hCG can be formed by different CGB subunits binding to CGA. One question that emerged with observing the different types of heterodimers was whether there are functional differences for the hCG variants. As hCG is well known for its substantial glycosylation, it was also debated whether functional differences between CGB7 and CGB3/5/8 could stem from varying glycosylation patterns. Because the O-glycans attached to the C-terminus of CGB are responsible for the significantly longer serum half-life of hCG compared to LH [32–34], differences in O-glycosylation between CGB7 and CGB3/5/8 might affect their serum half-lives and thereby affecting biological function. Even the usage of the same receptor is not necessarily proof for an identical biological function as was shown for hCG and LH which share a common receptor (LHCGR) but also carry out individual functions [35].

With this study, we were able to investigate the N- and O-glycosylation profiles separately for beta-hCG subunits CGB3/5/8 and CGB7 for the first time. In order to obtain separate preparations of the two proteins, recombinant protein was synthesized and expressed in HEK293 or HeLa cells. Following affinity purification, the proteins were analyzed separately by MALDI-TOF-MS for their glycosylation. N- and O-glycan structures of CGB3/5/8 and CGB7 were qualitatively similar. Only small differences were observed in terms of relative intensity in the spectra. The N-glycans of CGB7 were mostly of the biantennary type and were slightly less sialylated and more fucosylated than the ones of CGB3/5/8. Overall, we did not observe major differences in glycosylation patterns between CGB3/5/8 and CGB7. However, as expected, we observed differences between recombinant glycoproteins expressed in HEK293 and in HeLa cells. When expressed in HEK293 cells, N-glycans from CGB3/5/8 and CGB7 were carrying GalNAc-, core and antennary fucose, partial sialylation as we previously found on recombinant L-selectin [31] and on recombinant alpha-1 antitrypsin [36] expressed in HEK293 cells as well as on the surface of HEK293 cells [37]. On the contrary to L-selectin, GalNAc sulfation was not observed on N-glycans of CGB3/5/8 and CGB7 here (data not shown). This is probably due to the fact that SO_4_-GalNAc is rather an epitope observed on LH and TSH, which is a ligand of the macrophage mannose receptor [38]. The N-glycosylation pattern of CGB3/5/8 and CGB7 expressed in HeLa cells, an immortalized human cell line stemming from a primary cervical cancer, showed the presence of core- and antennary fucosylation. In addition, more sialylation was observed than for CGB3/5/8 and CGB7 produced in HEK293 cells. In our study, the major O-glycan structure was N1H1S2. Our data is in accordance with the N- and O-glycome of HeLa cells that is available in the database of the Functional Glycomics Gateway (Link: http://www.functionalglycomics.org).

In hCG isolated from the urine of pregnant women, biantennary (α2–3) disialylated core-fucosylated N-glycans constitute the majority of the structures [23, 24, 39] and traces of mono-antennary mono-sialylated and tri-antennary N-glycans can be found as well [23, 39, 40]. In malignancy, namely invasive mole and choriocarcinoma, hCG is composed of highly fucosylated and sialylated mono-, di- and tri-antennary N-glycans [24, 27, 28, 41]. High fucosylation is also observed in recombinant beta-hCG from CHO cells [25].

CGB7 contained slightly more core 1-type O-glycans and less core 2-type O-glycans than CGB3/5/8. Reports on pregnancy urinary hCG O-glycosylation were not systematically converging, which is probably due to site-specificity as well as to donor-dependency. At Ser 121, Valmu and coworkers found a majority of core-2 structures in urinary hCG whereas others reported core-1 structures [29]. At weeks 5 and 7 of pregnancy, core-2 structures were the highest and decreased with time. At Ser 138, Valmu *et al*. reported a majority of core-1 structures for hCG isolated from pregnant or molar disease patients whereas the disialylated core-2 structure was the most abundant peak in hCG from testicular cancer. The O-glycosylation sites at Ser 128 and 132 were reported to be occupied by core-1 and core-2 structures [24, 26, 39]. It should be noted that it is difficult to draw unambiguous conclusions for these sites because both of them were analyzed using a single glycopeptide. Indeed, there is no peptidic cleavage site between both O-glycosylation sites.

Regarding hCG functionality, a study by Kalyan and Bahl showed that deglycosylation of hCG did not affect subunit assembly or receptor binding, but resulted in impairment of its biological activity [32]. This indicates that glycosylation is important for hCG activity.

One important mechanism of hCG function is activation of the ERK1/2 pathway. Thus, we tested ERK1/2 activation induced by the different hCG variants. We chose the MAPK/ERK pathway as it can be activated by various upstream signaling events, intending to cover most of the known pathways described for hCG activity and not just the classical cAMP pathway. To this end, we created stably transfected cell lines to obtain recombinant proteins forming the two possible heterodimers of CGB with CGA. According to our knowledge, this is the first time that recombinant CGB7 was produced and compared to CGB3/5/8 concerning their biological activities. In previous studies investigating hCG activity, either recombinant hCG with only CGB3/5/8 or mixed hCG preparations purified *e.g*. from pregnancy urine were used. We showed that both dimer variants formed either by CGB7 or CGB3/5/8 with CGA are functional as they activate the ERK1/2 pathway. There was only a slight difference between CGB7 and CGB3/5/8 in the capability to induce ERK1/2 phosphorylation. Moreover, it was shown for the first time that hCG with CGB7 was also biologically active. Thus, hCG can also exert its functions in tissues where only CGB7 is expressed. Consistent with the ERK1/2 activation capability, we also found only minor differences in the glycan structures of the CGB variants.

These results leave open the question why humans can produce these two variants of hCG. It appears that CGB7 and CGB3/5/8 are functionally redundant isoforms. The difference in three amino acids between the two proteins does not lead to major differences in the functional assay that we applied. However, there are differences between the two gene groups in addition to the amino acid alterations they code for. While the promoter regions upstream of the coding parts of the *CGB3, CGB5* and *CGB8* genes display some degree of conservation, the promoter region of *CGB7* is clearly distinct from the other group of genes [22]. The differences in the promoter regions also lead to differential gene expression. We had shown that *CGB7* transcription can be induced by the p53 tumor suppressor while the other genes of the family are not [22]. The different promoter regions and the concomitant differential regulation is consistent with our observations that CGB7 but not CGB3/5/8 is expressed in various tissues like retina, urothelium, endometrium and decidua, whereas CGB3/5/8 is the dominant variant expressed by trophoblastic cells [5, 18–21].

Taken together, the results indicate that regulation of expression constitutes the primary difference between the two gene families. Therefore, our hypothesis is that CGB7 and CGB3/5/8 do not hold a significant functional difference but that timing and cell type of their expression is the key for understanding their divergent evolution.

## Materials and methods

### Plasmids

CGB7 and CGB3/5/8 expression constructs were created using cDNA from human placenta as template. Specific CGB cDNAs initially were amplified and cloned with *KpnI* and *XhoI* into the pcDNA3.1(+) vector (Invitrogen, Carlsbad, CA, USA) using the following isoform-specific primers: cDNA_CGB3/5/8_fwd CGG *GGT ACC* GGT ATA AAG CCA GGT ACA CG, cDNA_CGB7_fwd CGG *GGT ACC* GGT ATA AAG CCA GGT ACA CC and cDNA_CGB_rev CCG *CTC GAG* TTA TTG TGG GAG GAT CGG. The specific forward primers anneal upstream of the ATG in the 5’UTR as the sequences around the ATG could not be used to distinguish between the CGB variants. The resulting constructs were then used to subclone the respective coding sequences starting with ATG and missing the stop codon into the pcDNA3.1/myc-His(+)A vector (Invitrogen) using the primers CGB_CDS_fwd CGG *GGT ACC* ATG GAG ATG TTC CAG GGG CTG and CGB_CDS_rev AAAAA *ACC GGT* TTG TGG GAG GAT CGG GGT G. Cloning was performed using *KpnI* and *AgeI* restriction enzymes. Use of *AgeI* resulted in cutting out of the myc-tag so that the polyhistidine-tag (His-tag) is encoded in frame at the C-terminus of CGB.

CGA expression from the first construct we created with the obvious cloning from the first ATG to the stop codon resulted only in marginal CGA protein expression. Therefore, a construct similar to the “minigene” construct by Matzuk and Boime [42], who seemed to have faced the same problems in overexpressing CGA, was created in multiple steps. Finally, the CGA expression construct consisted of the cDNA from 5′ to 3’ UTR as well as intron 3. The construct was cloned into the pcDNA3.1(+)/Zeocin vector (Invitrogen) making use of *KpnI* and *XhoI* restriction sites. LHCGR expression vector (GFP-tagged) was obtained from Origene (Rockville, MD, USA, # RG222051).

### Cell culture

HEK293 cells (# ACC 305, DSMZ, Braunschweig, Germany) as well as HeLa cells (# ACC 57, DSMZ) were used for stable transfections. Both cell lines were cultivated in DMEM with 10% FCS. His-tagged CGB7- and CGB3-expressing stably transfected cell lines were obtained by transfection with the respective expression constructs in a pcDNA3.1/His vector. First, constructs were linearized using a *BglII* restriction site (30 min, 37 °C) and purified with a DNA cleanup kit from Qiagen (Hilden, Germany). Then, cells were transfected in 6-well plates (10^6^ cells per well were plated and cultivated for a day before transfection) with Fugene® HD transfection reagent (Promega, Madison, WI, USA) according to the manufacturer’s suggestions (1 μg vector and 2 μl Fugene® HD per well). For selection of transfected cells, 48 h after transfection, medium was changed to DMEM with Geneticin (0.6 mg/ml, Thermo Fisher Scientific, Waltham MA, USA). Mock transfected cells served as control to recognize the time when all non-transfected cells had died. To create single cell clones, 96-well-plates were prepared with 100 μl DMEM with Geneticin® per well. 20 μl of a cell suspension containing 150 cells/ml were added to each well. Only wells containing exactly one cell were further cultivated. Stably transfected cell pools as well as single cell clones were analyzed regarding their CGB glycan structures.

To obtain stable HEK293 cell lines producing both CGB and CGA in one cell, the CGB7- and CGB3-expressing stably transfected cells were transfected as described above with the CGA expression construct (in pcDNA3.1(+)/Zeocin). DMEM with Geneticin (0.6 mg/ml) and Zeocin (Thermo Fisher Scientific; 200 μg/ml) was used for selection. After all control cells had died, Zeocin concentration was reduced to 40 μg/ml. HEK293 cells stably expressing the LH/hCG receptor (LHCGR) were created as described above using Geneticin as selection agent.

For recombinant protein production, stably transfected cell lines were seeded into 300 cm^2^ cell culture flasks. After reaching confluence, cells were washed two times with PBS and cultivated for another 7 days in 30 ml CD293 medium (Thermo Fisher Scientific). After 7 days the cell culture supernatant containing CGB was harvested. Cells were removed by centrifugation and subsequent sterile filtration of the supernatant.

### Purification of recombinant CGB and hCG

His-tagged CGB7 or CGB3/5/8 was purified from cell culture supernatants by nickel-histidine affinity chromatography using Ni-NTA agarose beads (Qiagen) according to the manufacturer’s instructions. All steps were carried out on ice. 1 ml Ni-NTA agarose bead solution was used per 60 ml supernatant. Beads were washed two times with PBS and incubated with the supernatant for 90 min at 4 °C. The suspension was then given to a polypropylene column (5 ml, Qiagen, #34964) holding back the beads with bound CGB. Beads were washed on column three times with washing buffer 1 (PBS with 5 mM imidazole and 2.5% glycerol) and three times with washing buffer 2 (PBS with 10 mM imidazole and 3% glycerol) by incubating the beads with 13 ml of washing buffer for 5 min, 2 min or 1 min, respectively. His-tagged CGB was eluted from the beads with 2 ml of 250 mM imidazole in PBS for 15 min at room temperature. Three further elution steps with 1 ml elution buffer each were carried out to regain most of the bound CGB. Imidazole was removed from the eluates with Vivaspin®2 (Sartorius, Göttingen, Germany, #VS02H01) centrifugal concentrators according to the manufacturer’s instructions using half-concentrated PBS as washing buffer. Eluates were further concentrated using a vacuum concentrator.

Functional heterodimeric hCG (CGB + CGA) was purified from cell culture supernatants as described above. The recombinant CGA does not contain a His-tag itself but is co-purified with CGB-His, to which it is linked non-covalently.

### SDS-PAGE, Western blotting and hCG immunoassay

SDS-PAGE and Western blotting were performed using reducing conditions as described before (25). Gels with 12% (hCG subunit detection) or 10% acrylamide (ERK1/2 assay) were used, respectively. To control affinity purification efficiency, samples from each purification step were separated by SDS-PAGE and gels were stained with Coomassie Brilliant Blue (Bio-Rad Laboratories, Hercules, CA, USA) according to the manufacturer’s instructions. Specificity of CGB and CGA expression products was tested by Western blotting using our own customized isoform-specific polyclonal rabbit antibodies against CGB7 (1:250) and CGB3/5/8 (1:5000) [5] as well as rabbit anti-CGA antibody (1:500, Chemicon). Membranes were incubated with primary antibodies overnight at 4 °C. After washing three times with TBS-T, blots were incubated with goat anti-rabbit secondary antibody (1:5000, HRP-conjugated, Jackson ImmunoResearch Europe Ltd., Ely, UK, #111–035-144) for 1 h at room temperature. For detection SuperSignal™ West Dura Extended Duration Substrate (Thermo Fisher Scientific) was used.

For glycan analysis, purified CGB variants (24 μl of rebuffered and concentrated recombinant CGB with approx. 3000 U/ml hCG) were separated by SDS-PAGE (12% acrylamide) and stained with Coomassie Brilliant Blue.

Concentrations of hCG protein were determined employing the HCG + β Elecsys electrochemoluminescence immuno assay (Roche, #03271749190). According to the 3rd International Reference Preparation (International Standard 75/537) 9286 IU (international units) of hCG correspond to 1 mg.

For the ERK1/2 phosphorylation assay the following primary antibodies were used: rabbit anti-ERK1/2 (1:1000, Cell Signaling Technology, Frankfurt am Main, Germany, #4695), rabbit anti-phospho-ERK1/2 (1:1000, Cell Signaling Technology, #4370), and rabbit anti-GAPDH (Merck, Darmstadt, Germany, #G9545).

### In-gel N-glycan release

Coomassie-stained bands were excised using a spot cutter and N-glycans were released from the SDS gel pieces as described previously in Weiz *et al*. [43]. In short, gel pieces were covered with 25 mM phosphate buffer pH 7.1 containing 2 mg/L n-octyl-β-D glucopyranoside (Roth, Karlsruhe, Germany) and 2 U PNGase F asparagine amidase from *Flavobacterium meningosepticum* (EC 3.5.1.52, PNGase F, Roche Applied Science, Mannheim, Germany). Samples were incubated overnight at 37 **°**C. N-Glycans were extracted from the gel after incubation with acetonitrile at room temperature followed by Milli-Q water incubation at 50 **°**C. Eluates were pooled, desalted on self-made graphite tips [44] and permethylated.

### In-gel O-glycan release

Gel bands were incubated in 25% ammonia overnight at 45 **°**C. After the reaction, the solution was removed by centrifugal evaporation. Afterwards, 100 μl of 0.1% aqueous TFA was added and evaporated by centrifugal evaporation. Purification was performed using self-made microcolumns using a table centrifuge [45]. In short, 100 μl pipette tips equipped with a filter were filled with first with 40 μl SP20SS hydrophobic beads (Supelco, Bellefonte, PA, USA) then with 40 μl Dowex (50WX8–400, H+ Form; Sigma-Aldrich, St. Louis, MO, USA) that were previously conditioned as recommended by the manufacturer. After samples were applied, microcolumns were washed 3 times with 30 μl of 0.1% TFA and O-glycans were eluted 3 times with 30 μl 25% acetonitrile/0.1% TFA. O-Glycan samples were finally dried in a vacuum centrifuge.

### Permethylation

Permethylation of N- and O-glycan samples was performed using methyl iodide according to classical protocols [31]. After the reaction, samples were purified by liquid-liquid extraction using chloroform and water. Samples were finally dried by centrifugal evaporation and reconstituted in 75% acetonitrile.

### MALDI-TOF mass spectrometry

MALDI-TOF mass spectra were recorded on an Ultraflex III mass spectrometer (Bruker Daltonics) equipped with a Smartbeam laser and a LIFT-MS/MS facility using the reflector positive-ionization mode. 0.5 μl permethylated sample was mixed on a ground steel target in a ratio 1:1 with the matrix (10 mg/mL super DHB dissolved in 10% acetonitrile). Spectra consisted of at least 2000 shots. Calibration was performed on a dextran hydrolysate ladder. Processing of spectra was performed using Flexanalysis (Bruker Daltonics, Bremen, Germany). Glycan structures were assigned using GlycoPeakfinder.

### Statistical analysis

The Shapiro-Wilk-Test was used to test the normality of the considered sample group. Moreover, skewness and the kurtosis of the sample group were assessed in conjunction with the graphical representation of the distribution. As samples were not normally distributed, the Mann-Whitney U-test was used to assess the *p* value between two variables. *P* < 0.5 and *p* < 0.01 were considered to be statistically significant.

### ERK1/2 phosphorylation assay

231 million HEK293 cells stably transfected with LHCGR were seeded into cell culture dishes with 6 cm diameter (10^6^ cells per dish). 48 h later, after washing cells with PBS, 2 ml Opti-MEM™ medium (Thermo Fisher Scientific) were added per dish to starve the cells for 30 min at 37 °C. After 30 min, recombinant hCG was added in different concentrations and incubated for different times at 37 °C. As controls, recombinant LH (Luveris™, Merck Serono, Darmstadt, Germany) and recombinant commercially available hCG (Ovidrel®, Merck Serono) were used as well as recombinant single subunits and purified supernatants from HEK293 cells stably transfected with empty pcDNA3.1-His vector (see above). Following stimulation, cells were washed with PBS and immediately frozen in liquid nitrogen. All further steps were carried out on ice. Protein extraction was performed with 200 μl RIPA buffer per dish (including phosphatase and proteinase inhibitors). Cells were incubated for 5 min with RIPA buffer on ice. Then cells were scraped off the dish and transferred into an 1.5 ml tube. After homogenization by pipetting the lysate for 5 times, the cell lysate was centrifuged for 20 min (14,000 x g at 4 °C). The supernatants were analyzed by Western blotting determining phosphorylated ERK1/2 as well as total ERK1/2 and GAPDH. To this end, 1.5 μg of each lysate was separated by SDS-PAGE on 10% acrylamide gels. Densitometric analysis was carried out using the LabImage software (Kapelan Bio-Imaging, Leipzig, Germany).

## Electronic supplementary material

ESM 1 (PDF 3877 kb)
