# Supplementary material for: N- and O-glycosylation patterns and functional testing of CGB7 versus CGB3/5/8 variants of the human chorionic gonadotropin (hCG) beta subunit
Source: Glycoconj J. 2020 Aug 7;37(5):599–610. doi: 10.1007/s10719-020-09936-w (PMC7501100; doi:10.1007/s10719-020-09936-w)

Supplemental Fig. S1

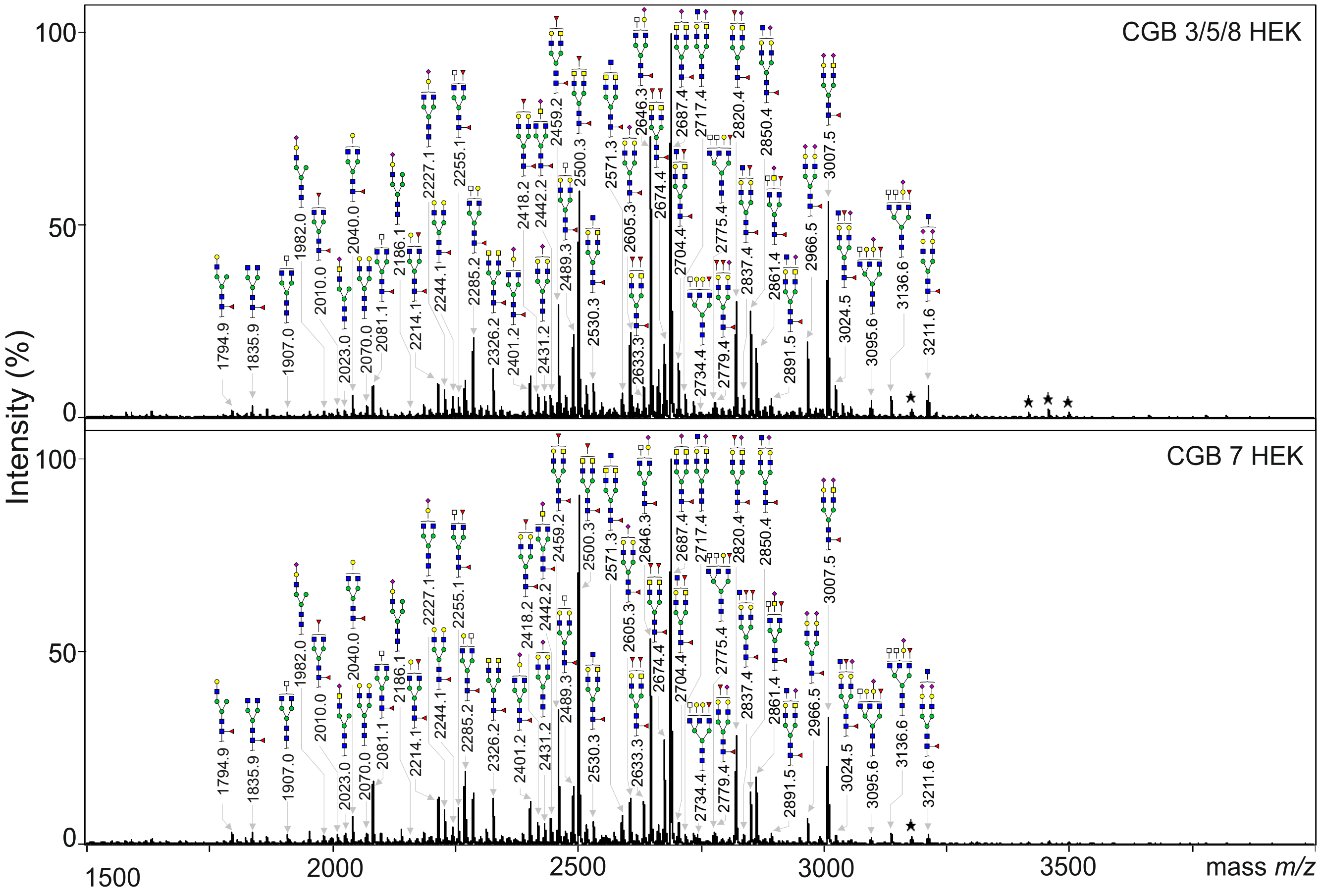

Supplemental Fig. S2

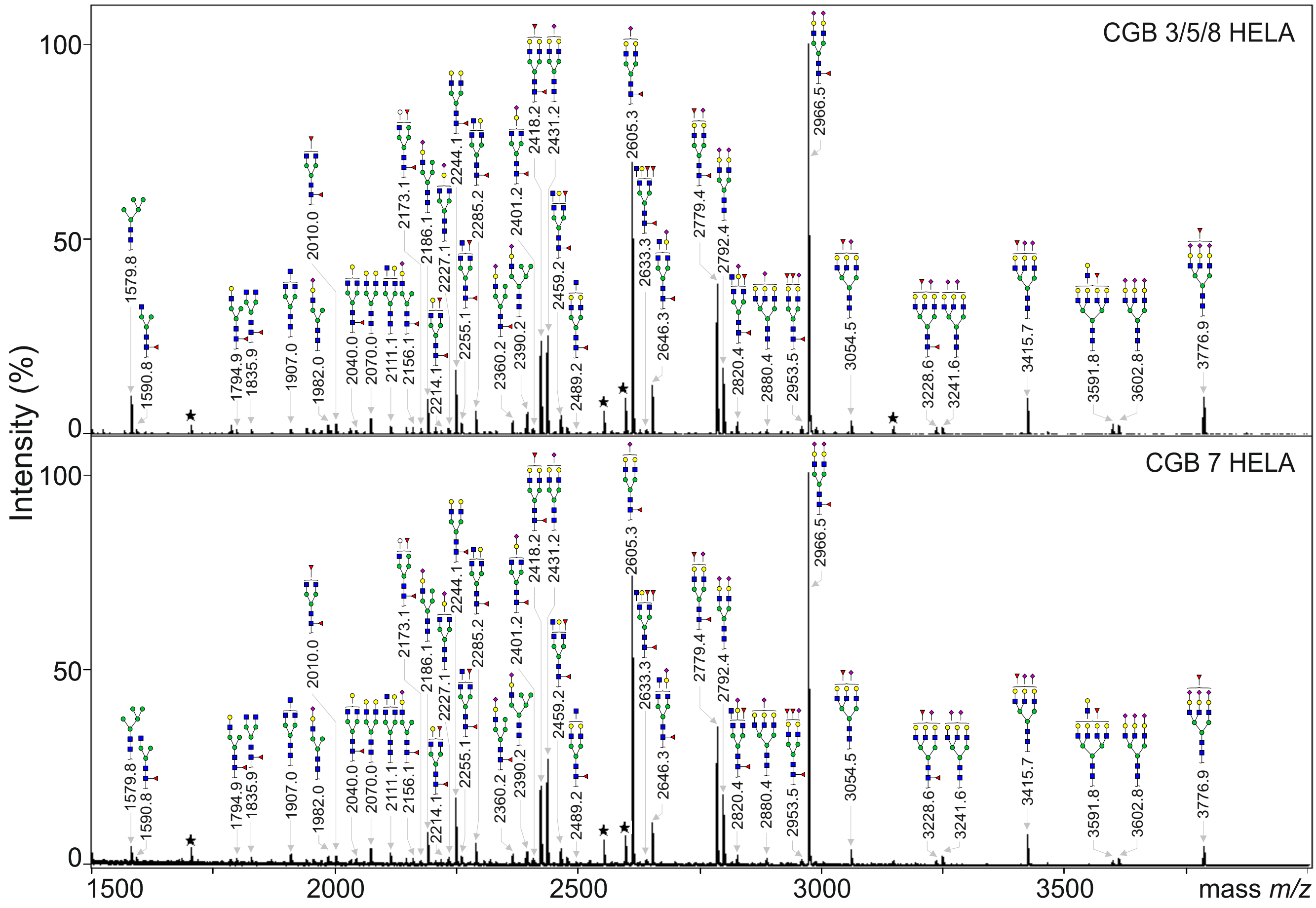

Supplemental Fig. S3

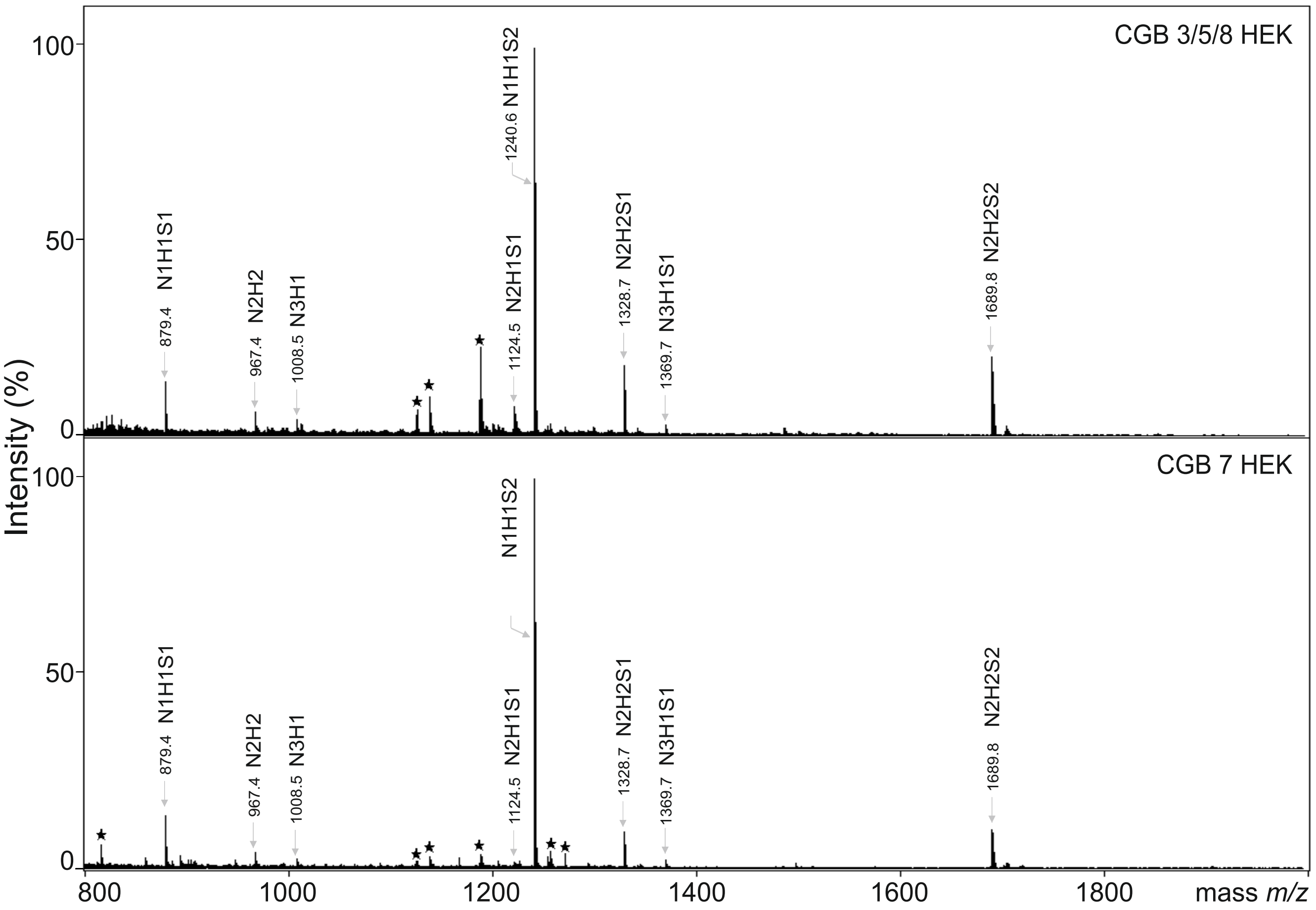

Supplemental Fig. S4

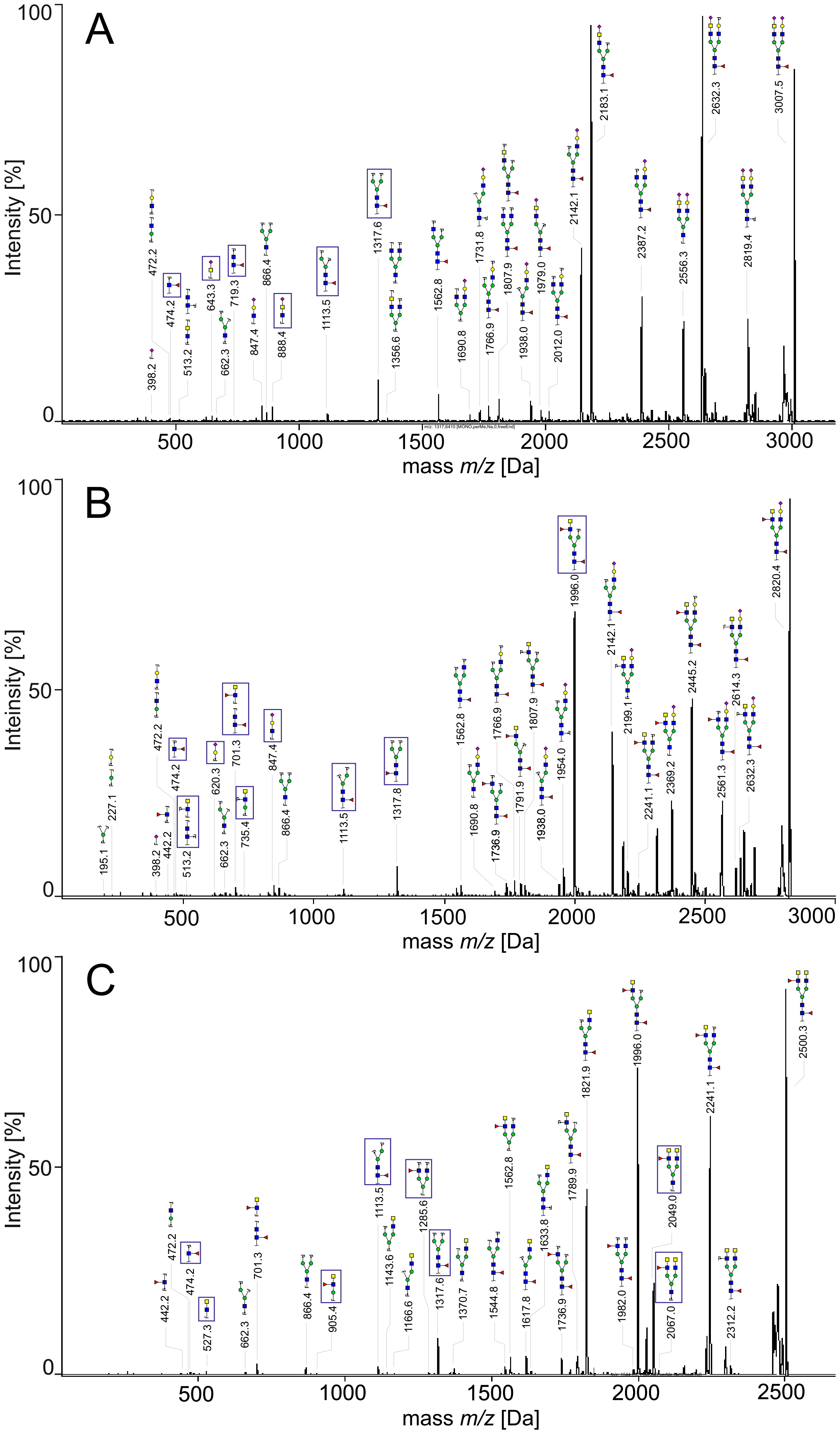

Supplemental Fig. S5

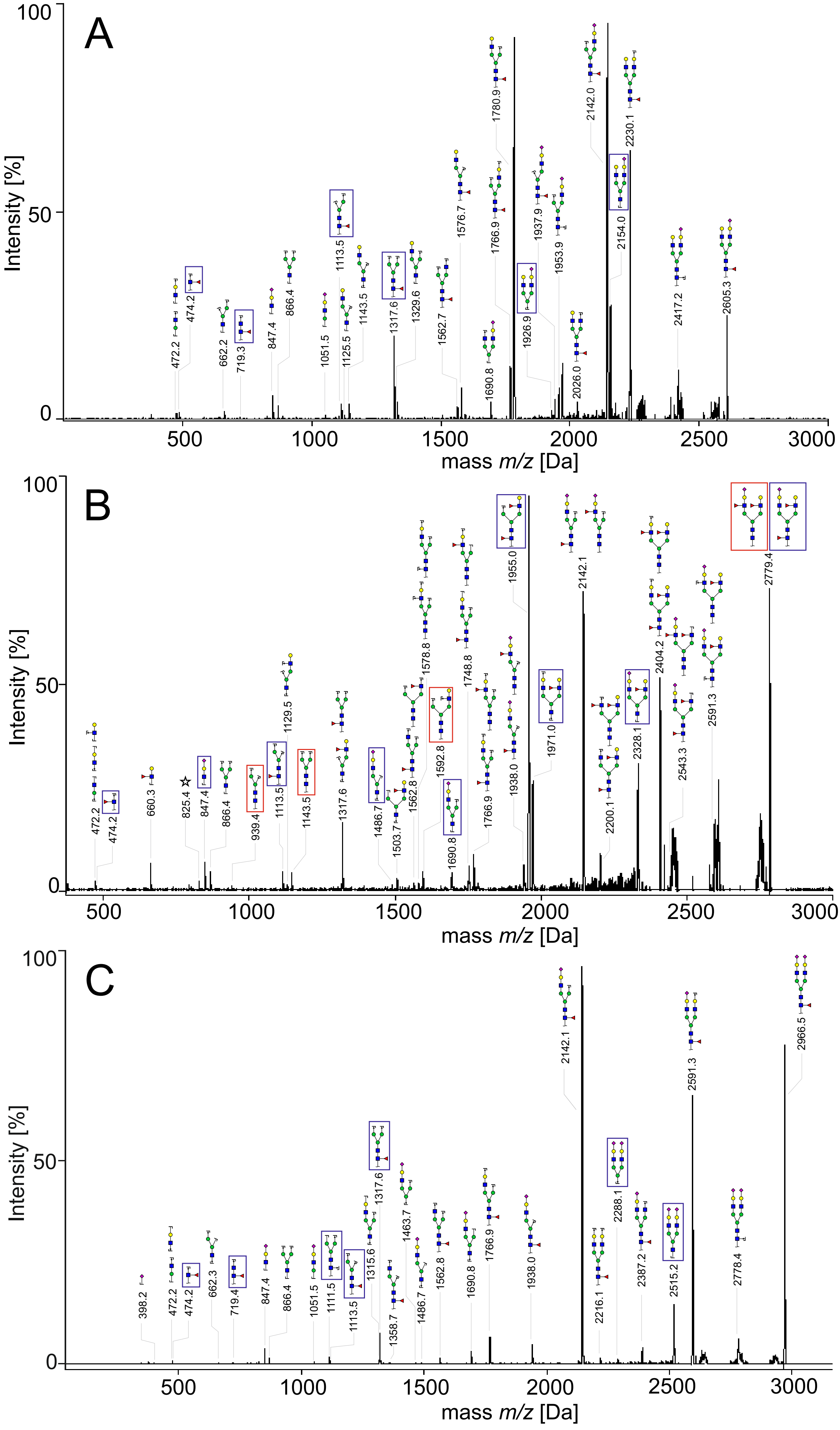

Supplemental Table S1

structure	composition	mass *m/z*	CGB 3/5/8 HEK	CGB 7 HEK	CGB 3/5/8 HELA	CGB 7 HELA
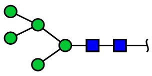	N2H5	1579.8	0.230	0.187	1.140	1.377
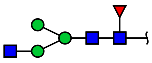	N3H3F1	1590.8	0.143	0.181	0.470	1.191
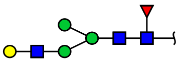	N3H4F1	1794.9	0.201	0.321	0.371	0.838
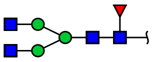	N4H3F1	1835.9	0.299	0.400	0.241	0.734
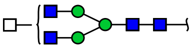	N5H3	1907.0	0.194	0.269	0.465	1.026
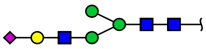	N3H4S1	1982.0	0.169	0.247	0.400	0.687
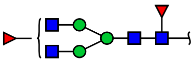	N4H3F2	2010.0	0.285	0.403	0.201	0.570
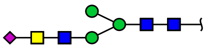	N4H3S1	2023.0	0.199	0.294	0.224	0.608
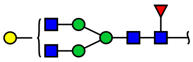	N4H4F1	2040.0	0.684	0.879	0.394	0.806
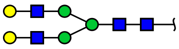	N4H5	2070.0	0.355	0.357	1.102	1.534
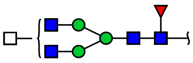	N5H3F1	2081.1	0.986	1.565	0.209	0.578
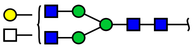	N5H4	2111.1	0.282	0.246	0.487	0.891
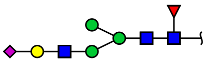	N3H4F1S1	2156.1	0.179	0.206	0.367	0.658
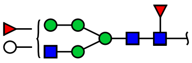	N3H5F2	2173.1	0.129	0.169	0.315	0.554
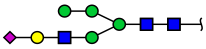	N3H5S1	2186.1	0.279	0.316	1.274	1.203
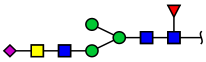	N4H3F1S1	2197.1	0.251	0.302	-	-

structure	composition	mass *m/z*	CGB 3/5/8 HEK	CGB 7 HEK	CGB 3/5/8 HELA	CGB 7 HELA
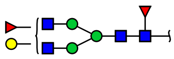	N4H4F2	2214.1	1.568	1.924	0.291	0.613
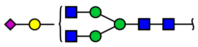	N4H4F1S1	2227.1	0.760	0.916	0.446	0.751
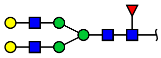	N4H5F1	2244.1	0.748	0.758	5.570	5.911
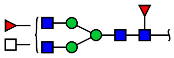	N5H3F2	2255.1	0.635	1.038	2.054	1.694
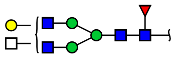	N5H4F1	2285.2	2.602	1.856	1.049	1.195
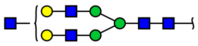	N5H5	2315.2	0.335	0.328	0.320	0.639
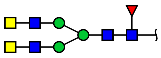	N6H3F1	2326.2	1.971	1.832	-	-
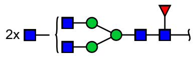			-	-	0.235	0.504
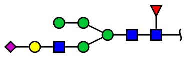	N3H5F1S1	2360.2	0.195	0.226	0.509	0.633
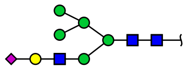	N3H6S1	2390.2	0.158	0.177	0.667	0.671
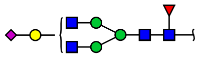	N4H4F1S1	2401.2	1.298	1.397	0.398	0.701
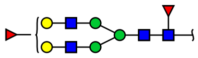	N4H5F2	2418.2	0.788	0.870	4.773	4.149
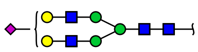	N4H5S1	2431.2	0.661	0.724	4.326	3.924
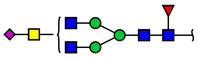	N5H3F1S1	2442.2	0.734	0.949	-	-
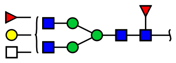	N5H4F2	2459.2	5.440	5.379	0.924	1.020
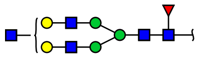	N5H5F1	2489.3	3.189	2.396	0.264	0.561

structure	composition	mass *m/z*	CGB 3/5/8 HEK	CGB 7 HEK	CGB 3/5/8 HELA	CGB 7 HELA
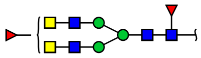	N6H3F2	2500.3	11.676	14.229	-	-
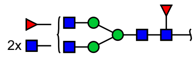			-	-	0.270	0.573
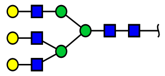	N5H6	2519.3	0.459	0.520	0.366	0.725
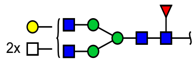	N6H4F1	2530.3	1.007	0.897	0.222	0.501
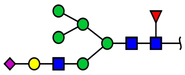	N3H6F1S1	2564.3	1.172	0.189	0.349	0.590
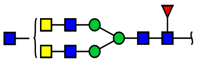	N7H3F1	2571.3	0.363	0.415	-	-
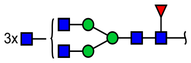			-	-	0.227	0.535
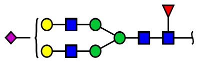	N4H5F1S1	2605.3	2.368	1.739	20.194	16.473
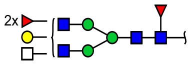	N5H4F3	2633.3	1.176	1.514	0.510	0.730
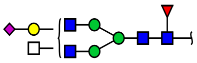	N5H4F1S1	2646.3	8.847	7.125	2.089	1.647
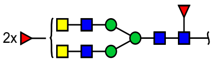	N6H3F3	2674.4	3.629	4.824	-	-
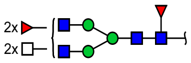			-	-	2.276	0.528
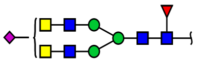	N6H3F1S1	2687.4	12.709	12.530	-	-
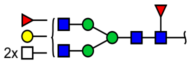	N6H4F2	2704.4	1.046	0.958	0.283	0.560
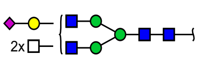	N6H4S1	2717.4	0.470	0.507	0.254	0.514
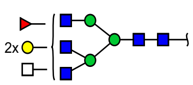	N6H5F1	2734.4	0.457	0.448	0.327	0.565

structure	composition	mass *m/z*	CGB 3/5/8 HEK	CGB 7 HEK	CGB 3/5/8 HELA	CGB 7 HELA
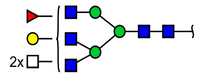	N7H4F1	2775.4	0.519	0.549	0.838	0.915
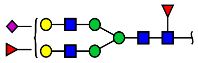	N4H5F2S1	2779.4	0.427	0.461	7.096	4.664
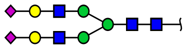	N4H5S2	2792.4	0.255	0.624	2.515	2.085
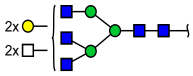	N7H5	2805.4	0.235	0.242	0.277	0.506
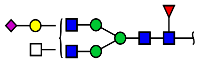	N5H4F2S1	2820.4	3.675	3.611	0.679	0.734
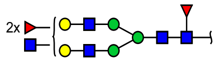	N5H5F3	2837.4	0.455	0.457	0.278	0.495
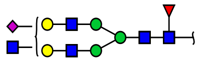	N5H5F2S1	2850.4	2.504	1.745	0.249	0.496
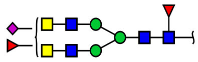	N6H3F2S1	2861.4	4.417	4.663	-	-
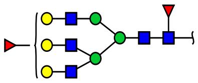	N5H6F2	2867.4	0.322	0.297	0.329	0.549
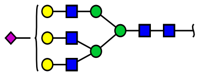	N5H6S1	2880.4	0.286	0.281	0.420	0.667
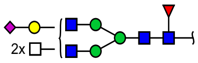	N6H4F1S1	2891.5	0.463	0.431	0.199	0.443
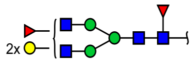	N6H5F2	2908.5	0.217	0.211	0.315	0.525
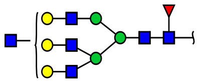	N6H6F1	2938.5	0.179	0.160	0.276	0.439
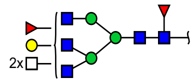	N7H4F2	2949.5	0.327	0.296	0.319	0.433
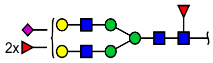	N4H5F3S1	2953.5	0.155	0.163	0.701	0.613
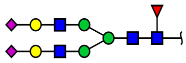	N4H5F1S2	2966.5	1.447	0.889	21.395	13.208

structure	composition	mass *m/z*	CGB 3/5/8 HEK	CGB 7 HEK	CGB 3/5/8 HELA	CGB 7 HELA
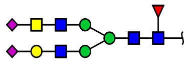	N5H4F1S2	3007.5	4.705	3.560	-	-
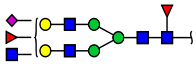	N5H5F2S1	3024.5	0.553	0.235	0.222	0.420
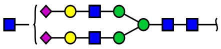	N5H5S2	3037.5	0.264	0.235	0.222	0.420
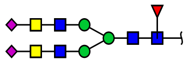	N6H3F1S2	3048.5	4.417	4.663	-	-
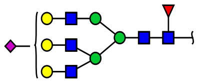	N5H6F1S1	3054.5	0.304	0.248	1.160	1.076
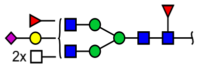	N6H4F2S1	3065.5	0.122	0.135	0.211	0.423
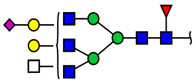	N6H5F1S1	3095.6	0.452	0.285	0.301	0.454
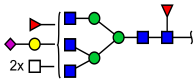	N7H4F1S1	3136.6	0.568	0.377	0.195	0.382
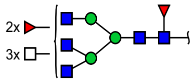	N8H3F3	3164.6	0.086	0.112	0.157	0.352
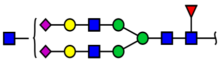	N5H5F1S2	3211.6	0.614	0.368	0.122	0.311
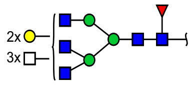	N8H5F1	3224.6	0.124	0.126	0.156	0.347
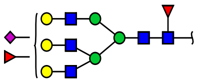	N5H6F2S1	3228.6	0.126	0.119	0.429	0.499
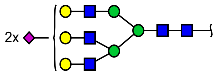	N5H6S2	3241.6	0.093	0.041	0.444	0.643
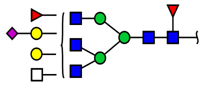	N6H5F2S1	3269.6	0.126	0.119	0.429	0.499
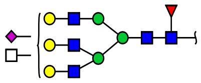	N6H6F1S1	3299.7	0.136	0.107	0.145	0.314
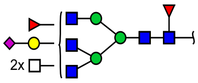	N7H4F2S1	3310.7	0.137	0.129	0.120	0.291

structure	composition	mass *m/z*	CGB 3/5/8 HEK	CGB 7 HEK	CGB 3/5/8 HELA	CGB 7 HELA
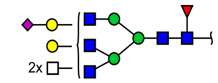	N7H5F1S1	3340.7	0.075	0.086	0.107	0.286
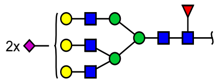	N5H6F1S2	3415.7	0.159	0.125	1.347	0.915
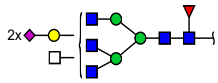	N6H5F1S2	3456.7	0.251	0.169	0.251	0.169
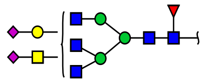	N6H4F1S2	3497.8	0.198	0.161	-	-
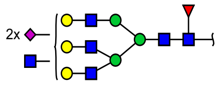	N6H6F1S2	3660.8	0.098	0.086	0.098	0.292
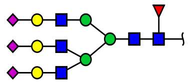	N5H6F1S3	3776.9	0.092	0.112	1.201	0.638
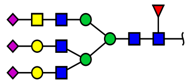	N6H5F1S3	3817.9	0.089	0.075	-	-

Supplemental Table S2

composition	mass *m/z*	CGB 3/5/8 HEK	CGB 7 HEK	CGB 3/5/8 HELA	CGB 7 HELA
N1H1S1	879.4	6.20	7.09	n.d.	n.d.
N2H2	967.4	3.58	2.84	n.d.	n.d.
N3H1	1008.5	2.80	2.12	n.d.	n.d.
N2H1S1	1124.5	5.21	2.70	n.d.	n.d.
N1H1S2	1240.6	55.20	67.12	n.d.	n.d.
N2H2S1	1328.7	10.36	6.80	n.d.	n.d.
N3H1S1	1369.7	2.34	2.13	n.d.	n.d.
N2H2S2	1689.8	14.29	9.19	n.d.	n.d.

Biskup, Blanchard et al. - Supplementary Information to Figures 1, 4 and 5 with unprocessed gels and blots

Supplementary Information to Figure 1C

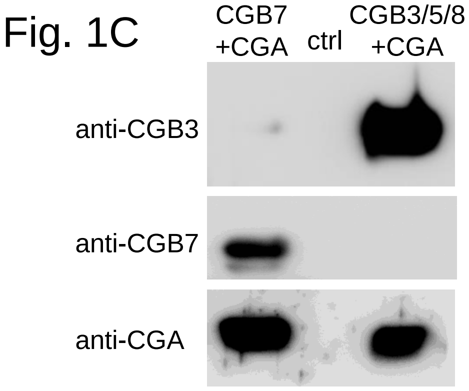

All samples were run on the same blot which was then cut and incubated with the respective antibodies. Left: less exposure time; right: longer exposure because the anti-CGB7 antibody is less sensitive than the other antibodies.

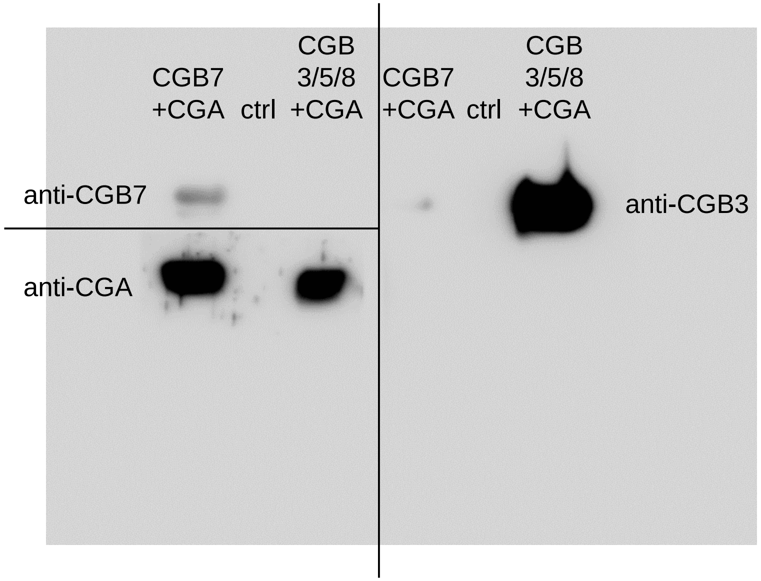

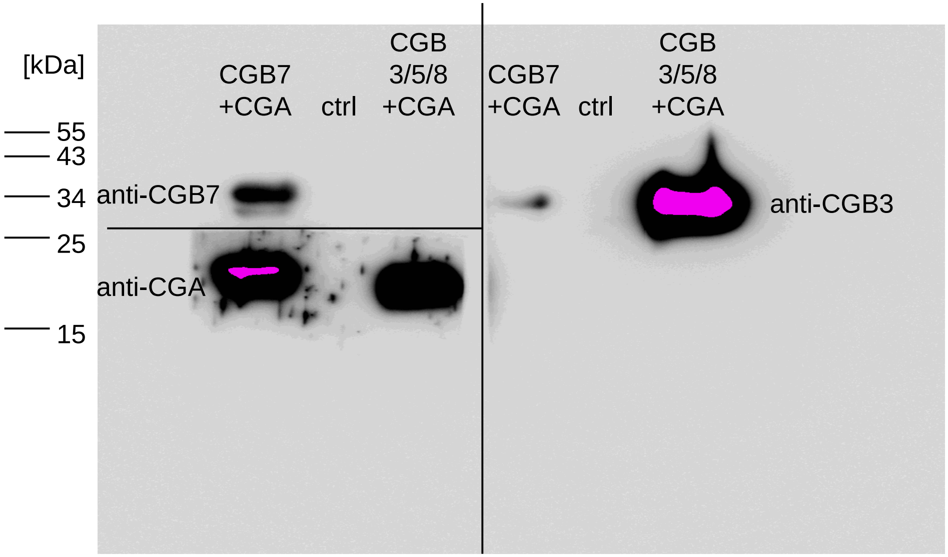

Supplementary Information to Figure 4

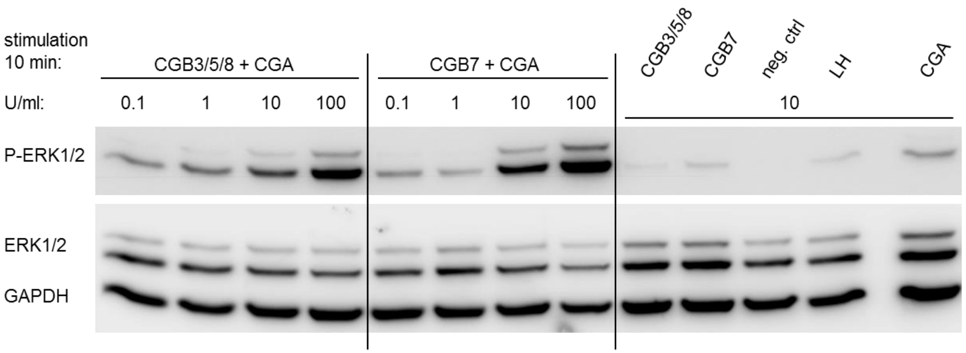

One blot was initially incubated with anti-P-ERK1/2 (upper graph), then stripped and incubated both with anti-ERK1/2 and GAPDH antibodies (lower graph).

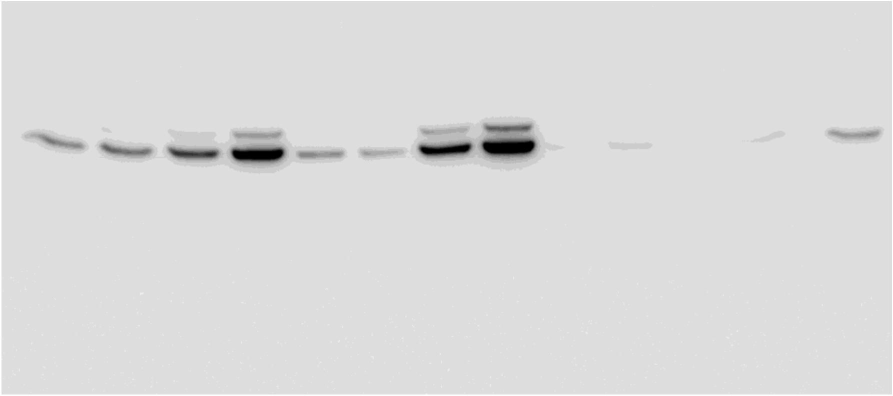

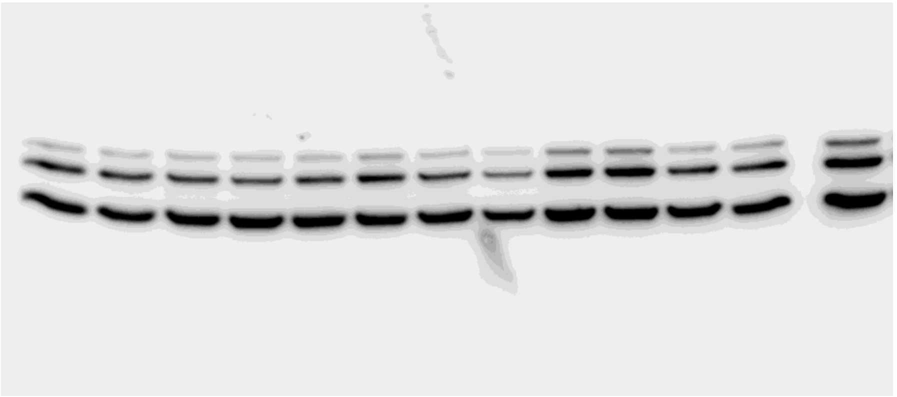

Supplementary Information to Figure 5

Two separate blots were processed in parallel from the same samples with the same quantities.

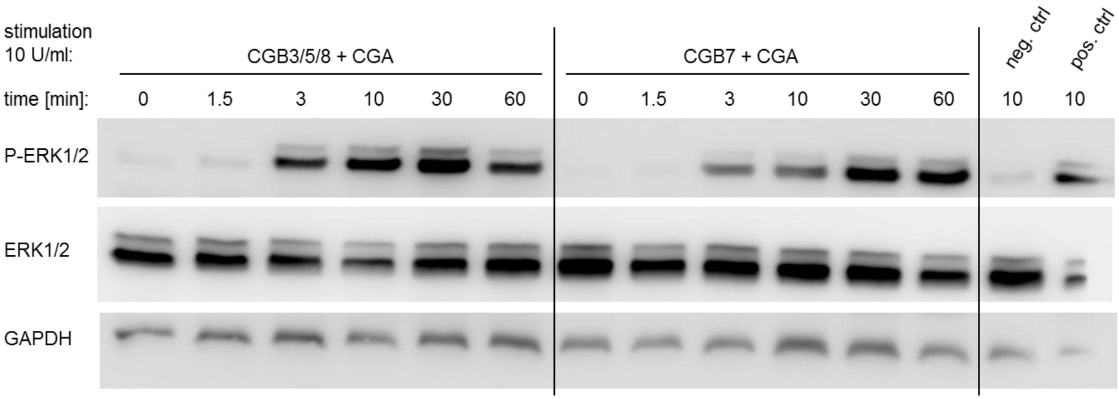

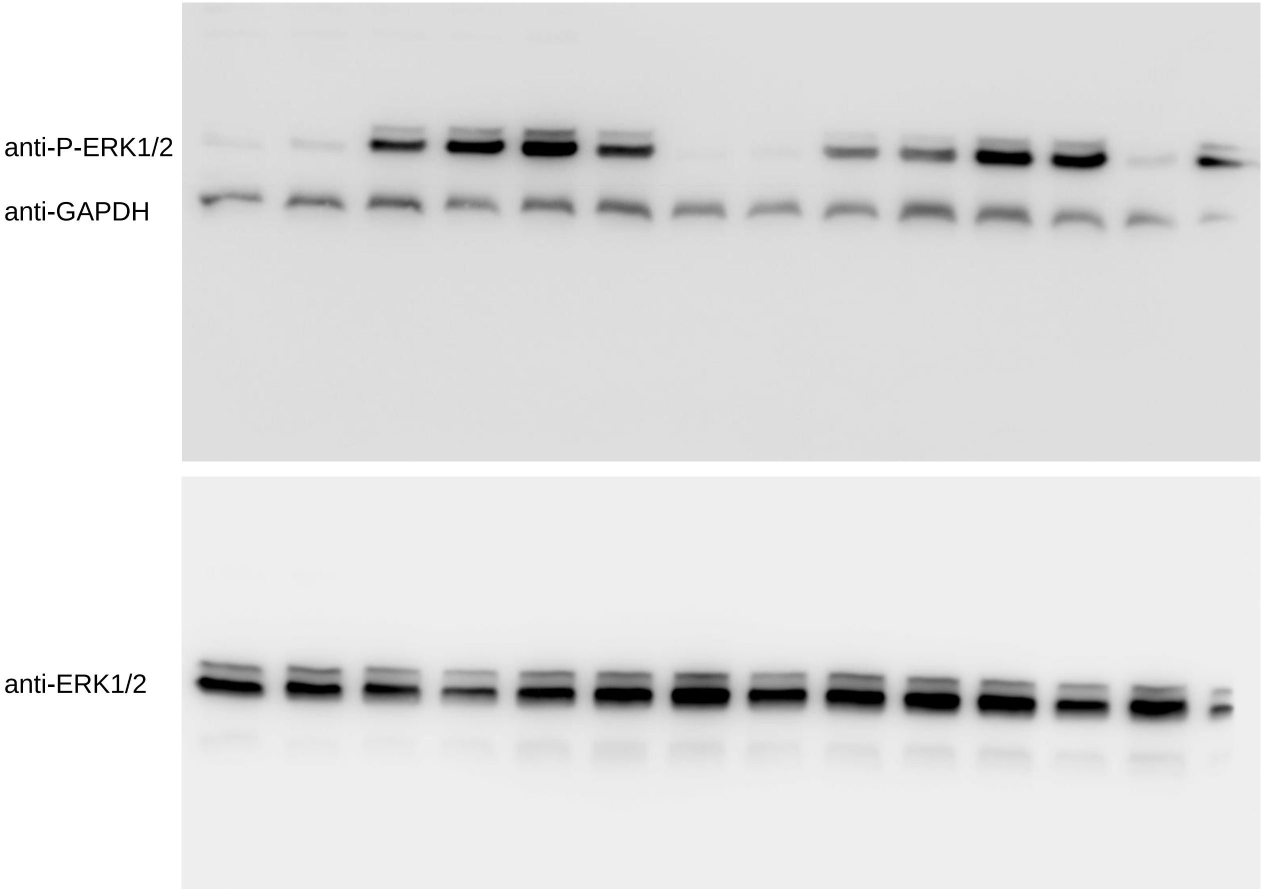

Supplement: Supplementary file 1 — (PDF 3877 kb) [file 10719_2020_9936_MOESM1_ESM.pdf]
